# Transition in Patients with Congenital Heart Disease in Germany: Results of a Nationwide Patient Survey

**DOI:** 10.3389/fped.2017.00115

**Published:** 2017-05-19

**Authors:** Paul C. Helm, Harald Kaemmerer, Günter Breithardt, Elisabeth J. Sticker, Roland Keuchen, Rhoia Neidenbach, Gerhard-Paul Diller, Oktay Tutarel, Ulrike M. M. Bauer

**Affiliations:** ^1^National Register for Congenital Heart Defects, Berlin, Germany; ^2^DZHK (German Centre for Cardiovascular Research), Berlin, Germany; ^3^Department of Paediatric Cardiology and Congenital Heart Disease, German Heart Centre Munich, Technical University of Munich, Munich, Germany; ^4^Division of Adult Congenital and Valvular Heart Disease, Department of Cardiovascular Medicine University Hospital of Münster, Münster, Germany; ^5^Competence Network for Congenital Heart Defects, Berlin, Germany; ^6^Department of Psychology, University of Cologne, Cologne, Germany; ^7^Bundesvereinigung JEMAH e. V., Aachen, Germany

**Keywords:** transition, congenital heart disease, online survey, adult with congenital heart disease, treatment, lost to follow-up, National Register for Congenital Heart Defects

## Abstract

**Background:**

A growing number of adults with congenital heart disease (ACHD) pose a particular challenge for health care systems across the world. Upon turning into 18 years, under the German national health care system, ACHD patients are required to switch from a pediatric to an adult cardiologist or an ACHD-certified provider. To date, reliable data investigating the treatment situation of ACHD patients in Germany are not available.

**Materials and methods:**

An online survey was conducted in collaboration with patient organizations to address the life situation and the conditions of health care provision for ACHD patients in Germany. ACHD patients were recruited from the database of the National Register for Congenital Heart Defects (NRCHD) and informed about the survey *via* email, websites, and social networks. A total of 1,828 ACHD patients (1,051 females) participated in this study. The mean age was 31.7 ± 11.7 years. Participants were surveyed about treating physicians and the institution mainly involved in the treatment of their CHD. In addition, participants were asked questions to assess the level of trust toward their treating physician and their familiarity with the term “ACHD-certified provider.”

**Results:**

Among the surveyed patients, 25.4% stated that they attended a specific ACHD clinic at a heart center regularly, 32.7% were treated in a private practice setting by a pediatric cardiologist, 32.4% in a private practice (adult) cardiology setting, and 9.5% were treated by an “other physician.” Only 24.4% of the male and 29.7% of the female ACHD patients were familiar with the term “ACHD-certified provider.”

**Conclusion:**

The transfer from pediatric cardiology to ACHD care requires further attention as many adult patients have not transferred to certified ACHD providers. The question of whether ACHD patients in Germany are offered consistent and adequate care should also be investigated in more detail. The answers regarding the ACHD certification are particularly disappointing and indicative of a large information gap and inadequate education in clinical practice.

## Introduction

About 6,000 children are born with congenital heart disease (CHD) in Germany each year (1). The number of adults with CHD (ACHD) is growing constantly (2, 3). Due to major advances of diagnosis and treatment, more than 90% of all children born with CHD reach adulthood today in western countries (2–6). The growing number of ACHD patients is a particular challenge for health care systems worldwide (7, 8). With increasing age, the medical needs of these patients are changing and motivate the need for a specific transition program (9). In this context, the term “transition” refers to the transitory stage from child-oriented care to a type of medical care that meets the requirements of adult patients (10).

Throughout their lives, patients with CHD have special medical and emotional needs. Thus, a successful transition from pediatric to adult centered care is particularly important (11, 12).

In 2010, the European Society of Cardiology (ESC) published practical guidelines for the management of ACHD (13). These guidelines, however, do not give specific recommendations regarding organization of ACHD care or further training for physicians (7, 13). It is accepted that ACHD patients have special needs requiring their treating physicians to have special expertise and training in the field of CHD in order to offer adequate care (4, 13–22). In Germany, certified heart centers and certified cardiologists/pediatric cardiologists in private practice, offering care tailored specifically to ACHD patients (23, 24) hold a certificate for “ACHD specialization.” The process for awarding “ACHD certification” has been standardized as a result of a cooperation within a joint task force that includes the German associations of cardiology, pediatric cardiology, and cardiothoracic/vascular surgery, as well as professional associations and patient organizations (23, 24).

Until their 18th year of life, patients are usually treated by pediatric cardiologists in private practice, heart centers, or university hospitals. According to the regulations of the German Medical Association, child and adolescent medicine, which includes pediatric cardiology, is responsible for treating infants, toddlers, children, and adolescents (25). The 19th year of life usually marks the end of adolescence (Youth Courts Law, par. 1, Social Act 8, par. 7 sections 1 and 2) (26, 27). As of this age, patients usually cannot be treated by a pediatrician any more.

Representative data regarding the question of who mainly treats ACHD patients in Germany for their CHD are not available. The present study aims to shed light on the treatment situation of ACHD patients in Germany and specifically answer the question of whether transition is successful or not. The answers to these questions are highly relevant not only in terms of adequate health care provision and policy but also for optimizing support for CHD patients and their relatives throughout their lives.

## Materials and Methods

The National Register for Congenital Heart Defects (NRCHD) conducted an online survey in collaboration with the two patient organizations “Bundesverband Herzkranke Kinder e. V.” (BVHK) and “Bundesvereinigung Jugendliche und Erwachsene mit angeborenem Herzfehler” (BV JEMAH). The survey’s primary objective was to collect information on the general life situation and the conditions of health care of ACHD patients in Germany.

With 51,134 members (as of October 2016), the NRCHD is Europe’s largest register of CHD. It is representative of the German cohort of patients with CHD (28). For patient recruitment, the register’s database was searched for patients who were 18 years or older at the time of the survey and for whom an email address was available. Respective individuals were invited to take part in the survey *via* email. In addition, the NRCHD, BVHK, and BV JEMAH informed ACHD patients about the survey *via* websites and social media channels.

Questions asked included:

- Which kind of physician mainly treats you for your heart disease?
- Do you attend regular follow-up examinations at a heart center/university hospital?
- Is the physician who mainly treats you for your heart disease ACHD-certified?
- Would you rather be treated by a pediatric cardiologist or an adult cardiologist?

Furthermore, four rating questions were asked using a six-tier scale for analysis:

- Do you understand the explanations given by your physician concerning your heart defect?
- Do you feel well-informed about your heart defect by your treating physician?
- How well do you rate your knowledge regarding your heart defect?
- How much do you trust your treating physician?

The six-tier scales were divided into three categories:

– 1−2 = low/negative rating
– 3−4 = medium/neutral rating
– 5−6 = high/positive rating.

For compiling the online questionnaire, the software EFS survey was used (29).

The respondents’ statements regarding their own CHD diagnosis were assigned to four groups according to Bethesda criteria (14).

The NRCHD has extensive experience in data collection *via* online surveys. The established data infrastructure of the NRCHD allows for storing data within the framework of an own data protection concept, which is registered with the Berlin Official for Data Protection and Freedom of Information (No. 531.390). General approval by the Ethics Committee Charité Berlin is available for all research conducted within the scope of the NRCHD. Registration to the NRCHD is voluntary. Participation is based on a broad consent. Patients agree that the NRCHD obtains and stores medical data from their attending physicians, for use in ongoing and future research studies until withdrawal. By consenting to this, patients have the option of taking part in studies and of regularly receiving information on the current state of research studies in the field of CHD *via* the patient website “www.herzregister.de.” The above Ethics Committee has approved the NRCHD ethical concept in 1999 and 2011. Participation in the NRCHD is promoted by patients’ and parents’ associations through their websites and in print.

### Statistical Analysis

The chi-square test was used for group comparisons including nominal data; data that were at least ordinally scaled were analyzed by using the Mann–Whitney *U* test or, in the case of more than two comparison groups, the Kruskal–Wallis test. Alpha error adjustment in multiple comparisons (30) was not performed as this was mainly an explorative and descriptive study and to avoid overlooking potential influencing factors.

SPSS (version 22) was used for all statistical analyses (31).

## Results

A total of 1,828 individuals participated. The mean age was 31.7 years (± 11.7 years) and 57.5% of patients were female (Table 1).

**Table 1:** **Sample composition (*N* = 1,828)**.

	% (*N*)	Age	Full-time employment	High education level	In a relationship
Total	100 (1,828)	31.7 ± 11.7	37.3% (681)	32.4% (592)	61.7% (1.128)
Male	42.5 (777)	32.7 ± 12.6	49.3% (383)	36.7% (285)	56.9% (442)
Female	57.5 (1,051)	31 ± 11	28.4% (298)	29.3% (307)	65.3% (686)
Simple congenital heart disease (CHD)	21.8 (398)	33.4 ± 14.2	40.2% (160)	34.2% (136)	67.3% (268)
Moderate CHD	33.2 (606)	31.1 ± 10.7	41.4% (251)	37.8% (229)	62.9% (381)
Complex CHD	38.2 (699)	32.2 ± 11.1	33.2% (232)	28.4% (199)	58.5% (409)
Unclassified CHD	6.8 (125)	26.7 ± 10	30.4% (38)	22.4% (27)	56% (70)

*N, sample size*.

### Underlying Heart Defect

At the beginning of the survey, the participants were asked to provide information regarding their CHD. Based on this information, patients were assigned to four diagnostic groups: simple CHD (*n* = 398), moderate CHD (*n* = 606), complex CHD (*n* = 699), and non-classifiable CHD (*n* = 125) (Table 1).

### Who Treats ACHD in Germany?

Overall, 58.1% of those surveyed were treated mainly at specific ACHD clinics at a heart center or by a pediatric cardiologist in private practice. Significant gender differences (*p* < 0.05) were found: women were more often treated at specific ACHD clinics at heart centers. Significant differences (*p* < 0.001) were also found between groups of CHD severity: In 70.5% of all cases, patients with complex CHD and in 43.5% of all cases, patients with simple CHD were treated mainly at a specific ACHD clinic at a heart center or by a pediatric cardiologist in private practice. More detailed information can be found in Table 2.

**Table 2:** **Descriptive statistics (subjective patient statements)**.

Which kind of physician mainly treats you for your heart disease?							
	**Total (*N* = 1,828)**	**Male (*n* = 777)**	**Female (*n* = 1,051)**	**Simple CHD (*n* = 398)^A^**	**Moderate CHD (*n* = 606)^B^**	**Complex CHD (*n* = 699)^C^**	**Others/unclassified CHD (*n* = 125)^D^**

Adults with congenital heart disease (ACHD) clinic at a heart center	25.4% (*n* = 465)	23.3% (*n* = 181)	27% (*n* = 284)	19.1% (*n* = 76)	23,1% (*n* = 140)	33,2% (*n* = 232)	13.6% (*n* = 17)
Pediatric cardiologist in private practice	32.7% (*n* = 598)	33.1% (*n* = 257)	32.4% (*n* = 341)	24.4% (*n* = 97)	34% (*n* = 206)	37,3% (*n* = 261)	27.2% (*n* = 34)
Adult cardiologist in private practice	32.4% (*n* = 592)	32.3% (*n* = 251)	32.4% (*n* = 341)	38.4% (*n* = 153)	36,8% (*n* = 223)	23,2% (*n* = 162)	43.2% (*n* = 54)
Another physician	9.5% (*n* = 173)	11.3% (*n* = 88)	8.1% (*n* = 85)	18.1% (*n* = 72)	6,1% (*n* = 37)	6,3% (*n* = 44)	16% (*n* = 20)
Group differences		***p < 0.05		**p < 0.001 (A vs. B: ***p < 0.001; A vs. C: ***p < 0.001; B vs. C: ***p < 0.001; B vs. D: ***p < 0.001; C vs. D: ***p < 0.001)			

**Do you attend regular follow-up examinations at a heart center/university hospital?**							

	**Total (*N* = 1,828)**	**Male (*n* = 777)**	**Female (*n* = 1,051)**	**Simple CHD (*n* = 398)^A^**	**Moderate CHD (*n* = 606)^B^**	**Complex CHD (*n* = 699)^C^**	**Others/unclassified CHD (*n* = 125)^D^**

At least once a year	53.8% (*n* = 984)	56.8% (*n* = 441)	51.7% (*n* = 543)	24.1% (*n* = 96)	54.1% (*n* = 328)	71.1% (*n* = 497)	50.4% (*n* = 63)
At least every 2 years	14% (*n* = 256)	12.7% (*n* = 99)	14.9% (*n* = 157)	16.8% (*n* = 67)	16.3% (*n* = 99)	10.3% (*n* = 72)	14.4% (*n* = 18)
At least every 3 years	6.4% (*n* = 117)	5.4% (*n* = 42)	7.1% (*n* = 75)	12.3% (*n* = 49)	6.9% (*n* = 42)	2.7% (*n* = 19)	5.6% (*n* = 7)
Less than every 3 years	20.1% (*n* = 368)	20.2% (*n* = 157)	20.1% (*n* = 211)	36.4% (*n* = 145)	18.2% (*n* = 110)	12.9% (*n* = 90)	18.4% (*n* = 23)
Never visited an ACHD-center	5.6% (*n* = 103)	4.9% (*n* = 38)	6.2% (*n* = 65)	10.3% (*n* = 41)	4.5% (*n* = 27)	3% (*n* = 21)	11.2% (*n* = 14)
Group differences		***p = 0.60		**p < 0.001 (A vs. B: ***p < 0.001; A vs. C: ***p < 0.001; A vs. D: *p* < 0.001; B vs. C: ***p < 0.001; C vs. D: ***p < 0.001)			

**Is the physician who mainly treats you for your heart disease ACHD-certified?**							

	**Total (*N* = 1,828)**	**Male (*n* = 777)**	**Female (*n* = 1,051)**	**Simple CHD (*n* = 398)^A^**	**Moderate CHD (*n* = 606)^B^**	**Complex CHD (*n* = 699)^C^**	**Others/unclassified CHD (*n* = 125)^D^**

Yes	27.8% (*n* = 509)	25.4% (*n* = 197)	29.7% (*n* = 312)	16.1% (*n* = 64)	24.8% (*n* = 150)	39.9% (*n* = 279)	12.8% (*n* = 16)
No	6.1% (*n* = 111)	5.1% (*n* = 40)	6.8% (*n* = 71)	6.3% (*n* = 25)	5.8% (*n* = 35)	7% (*n* = 49)	1.6% (*n* = 2)
I do not know	66.15% (*n* = 1208)	69.5% (*n* = 540)	63.6% (*n* = 668)	77.6% (*n* = 309)	69.5% (*n* = 421)	53.1% (*n* = 371)	85.6% (*n* = 107)
Group differences		*p < 0.05		*p < 0.001			

**Would you rather be treated by a pediatric cardiologist or an adult cardiologist?**							

	**Total (*N* = 1,828)**	**Male (*n* = 777)**	**Female (*n* = 1,051)**	**Simple CHD (*n* = 398)^A^**	**Moderate CHD (*n* = 606)^B^**	**Complex CHD (*n* = 699)^C^**	**Others/unclassified CHD (*n* = 125)^D^**

Pediatric cardiologist	28.5% (*n* = 521)	25.7% (*n* = 200)	30.5% (*n* = 321)	16.6% (*n* = 66)	25.7% (*n* = 156)	38.5% (*n* = 269)	24% (*n* = 30)
Adult cardiologist	30% (*n* = 549)	30.2% (*n* = 235)	29.9% (*n* = 314)	37.2% (*n* = 148)	32% (*n* = 194)	23.9% (*n* = 167)	32% (*n* = 40)
I do not know the difference	7.9% (*n* = 145)	9.7% (*n* = 75)	6.7% (*n* = 70)	9% (*n* = 36)	8.6% (*n* = 52)	6% (*n* = 42)	12% (*n* = 15)
I do not care	33.5% (*n* = 613)	34.4% (*n* = 267)	32.9% (*n* = 346)	37.2% (*n* = 148)	33.7% (*n* = 204)	31.6% (*n* = 221)	32% (*n* = 40)
		*p < 0.05		*p < 0.001			

*N, sample size*.

*Chi−squared test was used for the statistical analyses.

**Kruskal−Wallis test was used for the statistical analyses.

***Mann−Whitney U test was used for the statistical analyses.

### Regular Follow-up at a Specialized ACHD Clinic at a Heart Center

The majority of respondents (53.8%) stated that they attend a specific ACHD clinic at a heart center for a follow-up examination at least once a year (Table 2). Significant gender differences were not detected. However, significant differences (*p* < 0.001) according to CHD severity were found: While 71.1% of patients with complex CHD attended a specialized ACHD clinic at a heart center at least annually, only 24.1% of patients with simple CHD did so (Table 2).

### ACHD Certification of the Mainly Treating Physician

The majority of respondents (66.1%) stated that they did not know if the physician mainly treating them for their CHD was ACHD-certified. Significant gender differences (*p* < 0.05) were found: 29.7% of the female participants possessed knowledge about the ACHD certification status of their mainly treating physician compared to 25.4% of the male participants. Also in this case, patients with complex CHD were best informed regarding the their physician’s ACHD certification status, with 39.9% possessing knowledge, compared to participants with less complex disease (Table 2).

### Patient Preferences for Particular Physicians

Overall, 28.5% of those surveyed stated a preference for being treated by a pediatric cardiologist in private practice. Out of these, only 55.7% are actually treated mainly by a pediatric cardiologist in private practice. Thirty percent would prefer a treatment by an adult cardiologist in private practice (see Table 2), while 58.3% of these are actually treated in this setting. Furthermore, 7.9% of those surveyed stated not to know the difference between pediatric cardiology and adult cardiology, while 33.5% did to not care who mainly treats them (Table 2).

Significant gender differences (*p* < 0.05) were found. Female participants rather preferred treatment by a pediatric cardiologist in comparison to male participants (30.5 vs. 25.7%). Furthermore, significant differences (*p* < 0.001) according to CHD severity were detected: patients with complex CHD preferred pediatric cardiologist in private practice more often (38.5%) than patients with simple CHD (16.6%) (Table 2).

### Patient Age and Type of Main Treating Physician

**Figure 1:**
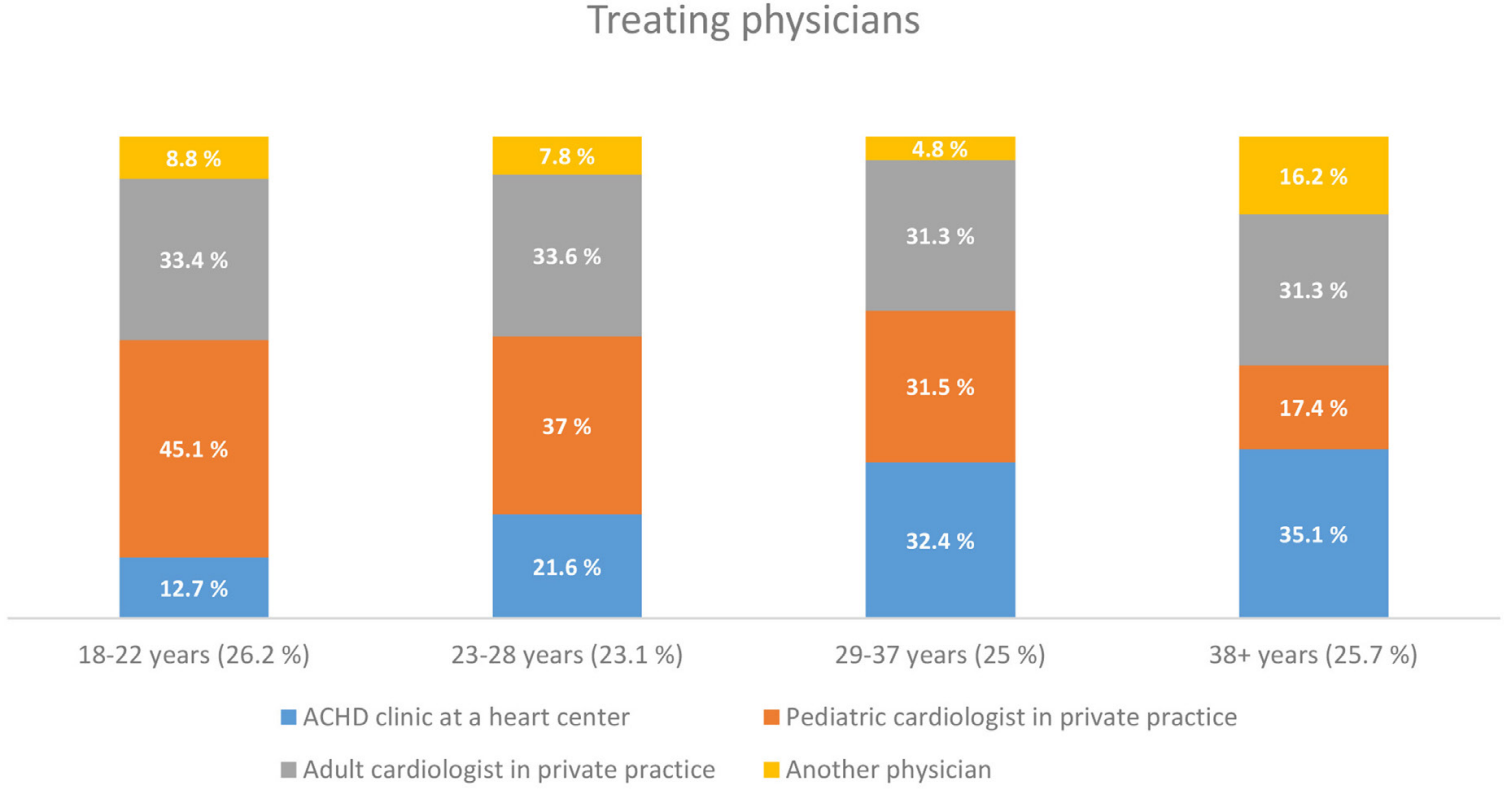
**Treating physicians**.

With increasing age, there was also a change regarding the type of main treating physician. While 45.1% of the respondents in the youngest age group (18−22 years) were treated mainly by a pediatric cardiologist in private practice, only17.4% of the respondents older than 38 years of age were treated in this setting (Figure 1).

### Rating Questions

Significant group differences were found regarding participants’ rating of the information about their CHD that they received by their main treating physician (*p* < 0.001). While only 64.7% of patients with CHD not treated in a specialist setting felt well well-informed about their condition by their physician, patients treated in a specialist setting more often felt well-informed (pediatric cardiologist in private practice: 79.4%, ACHD clinic at a heart center: 76.8%, adult cardiologist in private practice: 76.4%) (Table 3).

**Table 3:** **Descriptive statistics (subjective patient statements)**.

Do you understand the explanations given by your physician concerning your heart defect?					
	**Total (*N* = 1,828)**	**ACHD clinic at a heart center (*n* = 465)^A^**	**Pediatric cardiologist in private practice (*n* = 598)^B^**	**Adult cardiologist in private practice (*n* = 592)^C^**	**Another physician (*n* = 173)^D^**

Low	4% (*n* = 73)	3.7% (*n* = 17)	4% (*n* = 24)	4.2% (*n* = 25)	4% (*n* = 7)
Medium	17.6% (*n* = 325)	17.8% (*n* = 83)	16.9% (*n* = 101)	17.4% (*n* = 103)	22% (*n* = 38)
High	78.2% (*n* = 1430)	78.5% (*n* = 78,5%)	79.1% (*n* = 473)	78.4% (*n* = 464)	74% (*n* = 128)
Group differences		**p = 0.583			

**Do you feel well-informed about your heart defect by your treating physician?**					

	**Total (*N* = 1,828)**	**ACHD clinic at a heart center (*n* = 465)^A^**	**Pediatric cardiologist in private practice (*n* = 598)^B^**	**Adult cardiologist in private practice (*n* = 592)^C^**	**Another physician (*n* = 173)^D^**

Low	4.6% (*n* = 85)	4.1% (*n* = 19)	3.5% (*n* = 21)	4.9% (*n* = 29)	9.3% (*n* = 16)
Medium	19% (*n* = 347)	19.1% (*n* = 89)	17.1% (*n* = 102)	18.8% (*n* = 111)	26% (*n* = 45)
High	76.4% (*n* = 1396)	76.8% (*n* = 357)	79.4% (*n* = 475)	76.4% (*n* = 452)	64.7% (*n* = 112)
Group differences		**p < 0.001 (A vs. D: ***p < 0.01; B vs. D: ***p < 0.001; C vs. D: ***p < 0.01)			

**How well do you rate your knowledge regarding your heart defect?**					

	**Total (*N* = 1,828)**	**ACHD clinic at a heart center (*n* = 465)^A^**	**Pediatric cardiologist in private practice (*n* = 598)^B^**	**Adult cardiologist in private practice (*n* = 592)^C^**	**Another physician (*n* = 173)^D^**

Low	6.8% (*n* = 125)	5.4% (*n* = 25)	7% (*n* = 42)	6.9% (*n* = 41)	9.8% (*n* = 17)
Medium	38.8% (*n* = 710)	35.7% (*n* = 166)	41.5% (*n* = 248)	39.2% (*n* = 232)	37% (*n* = 64)
High	54.3% (*n* = 993)	58.9% (*n* = 274)	51.5% (*n* = 308)	53.9% (*n* = 319)	53.2% (*n* = 92)
Group differences		**p = 0.089			

**How much do you trust your treating physician?**					

	**Total (*N* = 1,828)**	**ACHD clinic at a heart center (*n* = 465)^A^**	**Pediatric cardiologist in private practice (*n* = 598)^B^**	**Adult cardiologist in private practice (*n* = 592)^C^**	**Another physician (*n* = 173)^D^**

Low	3.2% (*n* = 59)	2.4% (*n* = 11)	2.2% (*n* = 13)	3.7% (*n* = 22)	7.5% (*n* = 13)
Medium	16.8% (*n* = 307)	15.5% (*n* = 72)	13.2% (*n* = 79)	18.9% (*n* = 112)	25.4% (*n* = 44)
High	80% (*n* = 1,462)	82.2% (*n* = 382)	84.6% (*n* = 506)	77.4% (*n* = 458)	67.1% (*n* = 116)
Group differences		**p < 0.001 (A vs. D: ***p < 0.001; B vs. C: ***p < 0.01; B vs. D: ***p < 0.001; C vs. D: ***p < 0.01)			

*N, sample size*.

*Chi-squared test.

**Kruskal−Wallis test.

***Mann−Whitney U test.

Likewise, the question regarding participants’ trust in their treating physician yielded significant group differences (*p* < 0.001) (Table 3). The highest degree of trust was reported by those patients who were mainly treated by a pediatric cardiologist (Table 3).

## Discussion

According to the German health care system, patients with CHD may not generally be treated by a pediatric cardiologist in private practice once they have turned 18 years (25). The present study shows indeed, that, with increasing age, patients are less likely to be treated by a pediatric cardiologist in private practice. However, as many as 17.4% of the patients older than 38 years are still treated by a pediatric cardiologist in private practice. Transition from pediatric to an age-appropriate adult medical care, as defined by the transition concept (9, 10), can, therefore, be considered as being only partially successful in Germany based on these results. Adult patients with more complex underlying heart disease are those being mainly treated in a specific ACHD clinic at a heart center or by a pediatric cardiologist in private practice. The relatively large proportion of patients continuing to attend general cardiologists, not specializing in ACHD, supports the concept to provide additional training for adult cardiologists in the field of CHD. To this end, a process of ACHD certification (24) was established in Germany based on recommendations for improving the quality of the interdisciplinary care for ACHD (23). The main intention was to enable, both, pediatric cardiologists to treat adult patients, as well as to provide adult cardiologists with training and experience in the treatment of complex CHD. It is hoped that this addresses the challenges associated with the continuously growing and aging group of CHD patients (14) and ensure that patients receive the necessary support and medical care throughout their lives (24). The fact that a large majority of the surveyed patients did not know the meaning of the term “ACHD-certified,” as well as their ignorance regarding the fact of whether their treating physician is actually ACHD-certified, which shows that this certification measure is not appreciated and understood as a quality criterion by many patients. Therefore, despite the fact that especially patients with complex CHD prefer treatment at specialized ACHD centers, the question of whether the treating physician is ACHD-certified seems to play only a marginal role in choosing a particular center/physician.

One may also question the obligatory shift from pediatric to adult cardiology care in the German health care system. The main problem is that it may prevent a pediatric physician from caring for a patient known to him/her since the patient’s early childhood, just because of an age limit that could be regarded by some as arbitrary. This could lead to patients being less compliant with their care. This may be one possible explanation for the major problem of ACHD patients being “lost to follow-up” (32, 33). On the other hand, advocates of the transition system rightly argue that adults with CHD have very different needs from children with the conditions requiring particular expertise and training on behalf of the main treating (pediatric-) cardiologist. Resolving the question on the optimal organization of care for ACHD patients is beyond the scope of the current report, but our study provides important insights into the current status of treatment, patient education, and patient views on this topic in a contemporary cohort of German ACHD patients.

### Limitations

Since this is a cross-sectional study, we provide descriptive information and report on associations rather than claiming to report causal relationships between parameters. Moreover, the results reflect respondents’ subjective statements. The results may not be applicable to patients outside Germany, since they are affected by the life situation of the patients, as well as the organization of the health care system.

One might assume that patients registered in the NRCHD have a greater interest in CHD and therefore know more about this condition than German patients who are not registered.

Since the CHD patients have been invited to participate in the survey by emails, *via* websites and social networks, no reliable response rate can be specified. Therefore, we cannot guarantee that the sample of patients participating in the online survey is representative for the ACHD community at large. However, a previous study has showed that the patient population included in the register is representative. In addition, by involving large national patient organizations, we aimed to increase the reach of the survey and thus also capture patients not necessarily linked to major heart center. This should reduce bias related to more complex patients tending to be more likely associated to tertiary care and thus included in the register.

## Conclusion

Reassuringly, ninety percent of the participants were treated by medical specialists. Many patients made use of specific ACHD clinics at a heart center or were seen by pediatric/adult cardiologists in private practice. However, a sizeable proportion of patients was found to not being linked to recognized ACHD specialists, with approximately one-third of all respondents not in continuous medical care at a specific ACHD clinic/heart center. The trust in the treating physician seems to play a significantly more important role for the surveyed patients than an existing ACHD certification. Overall, there is still a major need for improvement of the (medical) care of ACHD patients.

## Author Contributions

PH, HK, GB, ES, RK, RN, G-PD, OT, and UB took responsibility for all aspects of the reliability and freedom from bias of the data presented and their discussed interpretation.

## Conflict of Interest Statement

The authors declare that the research was conducted in the absence of any commercial or financial relationships that could be construed as a potential conflict of interest.
